# Word onset tracking in neural responses of human basal ganglia nuclei

**DOI:** 10.1007/s00429-025-02968-8

**Published:** 2025-06-25

**Authors:** Arkan Al-Zubaidi, Inga M. Schepers, Anne-Kathrin Beck, Kerstin Schwabe, Joachim Runge, Mahmoud Abdallat, Joachim K. Krauss, Jochem W. Rieger

**Affiliations:** 1 Applied Neurocognitive Psychology Lab, Oldenburg University, Oldenburg, Germany; 2 Cluster of Excellence Hearing4all, Oldenburg University, Oldenburg, Germany; 3 Department of Neurosurgery, Hannover Medical School, Hanover, Germany; 4 Institute of Legal Medicine, Hannover Medical School, Hanover, Germany; 5 Department of Neurosurgery, University of Jordan, Amman, Jordan

**Keywords:** Local field potentials, Deep brain stimulation, Envelope of beta-band oscillations, Speech processing

## Abstract

Auditory speech is a highly relevant ecological stimulus with intrinsic temporal dynamics. At the cortical level, it is well established that neural responses contain information about the temporal dynamics of speech. Previous research further suggests that the basal ganglia also play a role in temporal processing. However, it is unknown whether neural responses in basal ganglia structures contain information about temporal speech structure. To address this question, we recorded local field potentials from the subthalamic nucleus (STN) and the globus pallidus internus (GPi) through contacts of deep brain stimulation (DBS) electrodes in patients with movement disorders (STN in Parkinson’s disease, GPi in Tourette syndrome or dystonia) while they listened to two-speaker speech streams with a task-relevant and an -irrelevant speech stream. Compared to baseline, amplitude modulations occurred in the beta-band (12–30 Hz) during listening to speech (word onset and duration) both in STN or GPi. Furthermore, the neural responses in both subcortical structures tracked word onsets during two-speech stream presentations, indicating that they code information about temporal speech structure. These findings provide further evidence that basal ganglia are involved in gating information about temporal speech structure relevant for task goal-directed processing.

**Supplementary Information:**

The online version contains supplementary material available at 10.1007/s00429-025-02968-8.

## Introduction

Human speech is characterized by its dynamic temporal structure, which is reflected in the activity of cortical neural dynamics (Ding et al. 2015; Kayser et al. 2015; Kubanek et al. 2013; Nourski et al. 2009; Peelle et al. 2013; Golumbic et al. 2013; Zoefel et al., 2022; Tankus et al., 2012). Despite significant degradation of other features, speech remains intelligible due to its temporal structure (Shannon et al. 1995; Golumbic et al. 2012). While speech perception primarily involves cortical processes such as auditory feature extraction (in the auditory cortex) and integration with morphosyntactic information (in Broca’s and Wernicke’s areas; Fonteneau et al. 2015; Friederici 2002), there are more layers of complexity.

Beyond the cortex, the basal ganglia (BG) and their interconnected thalamus circuitry have emerged as additional players in speech processing (Lim et al. 2014; Schepers et al. 2017; Beck et al. 2020; Tankus et al. 2024a, b). Studies on patients with BG dysfunction who exhibit impairments in speech production and comprehension underscore the involvement of this circuitry in the complex domain of speech (Kempler and Lancker 2002; Volkmann et al. 1992; Silveri 2021; Tankus et al., 2019). However, the contributions of the BG to encoding information from multiple speech sources remain unclear. It is possible that the BG are involved in selecting relevant information from a complex auditory scene or in integrating information from different speakers.

The BG have been demonstrated to play a critical role in resolving competition among alternatives across motor, cognitive, and sensory domains (Albin et al. 1989; Alexander 1986; Bočková et al. 2011; Combs et al. 2015; Utter and Basso 2008; Van Schouwenburg et al. 2015; Brittain and Brown 2014; Schwartze et al. 2011; Singh et al., 2011; Beck et al. 2018), providing a basis for investigation of its involvement in speech processing. Recent insights have expanded our understanding of the BG’s contribution to temporal processing, rhythm, sub-lexical speech processing, and attentional gating (Al-Zubaidi et al. 2022; Grahn 2009; Krauzlis et al. 2014; Nozaradan et al. 2017; Schubotz 2001; Van Schouwenburg et al. 2015; Wiener et al. 2014). Pathological disturbances in time perception associated with BG disorders further underscore its role in temporal processing (Allman and Meck 2012; Schwartze et al. 2011, 2015).

This expanding body of evidence, including functional magnetic resonance imaging (fMRI) findings that demonstrate BG activation during regular temporal stimulation (Geiser et al. 2012; Grahn 2009) and electroencephalogram (EEG) studies that reveal altered tone sequence cortical processing in patients with BG lesions (Nozaradan et al. 2017; Schwartze et al. 2015), highlights the essential role of the BG in cognitive control and executive processes, particularly in manipulating sequence information (Moore et al. 2013; Macoir et al. 2013). Furthermore, the responsiveness of the BG to regular temporal stimulation in both non-musicians and musicians (Grahn and Brett 2007; Grahn 2009) underscores its importance in processing temporal structures. Therefore, it is consequential to explore whether the BG encodes information about the temporal structure of speech in order to elucidate the neural mechanisms underlying speech processing (Albin et al. 1989; Alexander 1986; Bočková et al. 2011; Combs et al. 2015; Utter and Basso 2008; Van Schouwenburg et al. 2015).

The precise role of the BG in processing temporal speech structure within complex auditory scenes, such as two-speaker speech streams, remains unclear (Lim et al. 2014), primarily due to the limited spatial and temporal resolution of non-invasive neuroimaging techniques like EEG, MEG, and fMRI. Despite these challenges, clinical studies of patients with Parkinson’s disease (PD) and other movement disorders have leveraged direct and invasive brain measurements, such as deep brain stimulation (DBS) electrodes implanted in the subthalamic nucleus (STN), the globus pallidus internus (GPi) and other structures (Lozano et al. 2019; Krauss et al. 2021). These invasive methods provide insights that complement non-invasive techniques, highlighting the BG’s role in motor control and selection processes (Klostermann et al. 2008; Beck et al. 2018; Williams et al. 2014). The connection between these findings and the BG’s involvement in speech processing has been further supported by studies on challenging auditory conditions (Barnaud et al. 2018; Skipper and Hasson 2017). The integration between motor and auditory cortices aligns with the intricate interplay between the motor cortex, responsible for motor recruitment, and the BG, known for their role in motor selection and initiation (Hikosaka et al. 2000; Nambu 2004; Turner & Desmurget 2010), and it furthermore underscores the multifaceted role of the BG, serving as a bridge between motor control mechanisms and the intricate landscape of speech fluency.

The present study investigates the BG structures to track word onsets during the complex process of speech processing. Direct recordings of neural dynamics from the human STN and GPi via contacts of DBS electrodes have been shown to provide insight into the subcortical structures involved in sensory information processing (Airaksinen et al. 2011; Alam et al., 2015 ; Münte et al. 2017). We first acquired local field potential (LFP) recordings from the STN and the GPi of patients implanted with DBS electrodes while they listened to speech streams of two simultaneous speakers and performed a task focused on one of the speech streams. Then, word onset information from both speakers was extracted to determine each speech stream’s temporal structure. Prior research has demonstrated that word onsets facilitate lexical access and guide attention during speech processing (Astheimer & Sanders, 2009) ; Li et al. 2014, 2017; Sanford et al. 2006). Finally, we utilized machine learning techniques to estimate the temporal response function (TRF; Crosse et al. 2016) to analyze linear temporal relations between the word onsets in the two speech streams and the neural signal. In order to obtain an empirical p-value for individual electrode contact, a nonparametric bootstrap technique was employed to ascertain the statistical significance of the correction coefficient between predicted and actual neural signals. This was achieved by resampling the stimulus feature vector with replacement 100 times for each speech stream. This data-driven approach is further supported by recent advances demonstrating that speech-related neuronal activity in subcortical structures such as the STN and thalamus can be decoded using machine learning methods, allowing for inference of speech content at the single-neuron level (Tankus et al. 2021; Tankus et al., 2024a).

Although studies on the BG have indicated its involvement in temporal speech processing, it remains unclear whether BG structures specifically track word onsets during speech processing (Lim et al. 2014). Establishing a direct link between the neural dynamics of the STN, GPi, and the temporal structure of speech would provide compelling evidence for their role in this critical aspect of auditory processing. Therefore, the present study aims to explicitly test the hypothesis that BG structures, particularly the STN and GPi, actively contribute to tracking word onsets during speech processing.

## Materials and methods

### Participant information

The study was approved by the ethics committee of Hannover Medical School and informed consent was obtained from all participants. All participants were native German speakers. Nine patients suffering from PD who were implanted bilaterally in the STN participated in the experiment. One of the patients was removed from data analysis due to technical artifacts, resulting in eight STN data sets (STN1-STN8; 1 woman, age range 36–66, mean age 52, SD 11 years; seven participants were right-handed and one participant was ambidextrous). In addition, six patients suffering from either Tourette syndrome (GPi1-GPi3) or dystonia (GPi4-GPi6) were implanted bilaterally in the GPi (2 women, age range 19–59, mean age 38, SD 18 years; five participants were right-handed and one participant was left-handed) participated in the experiment. Demographic data of the individual patients are presented in Table 1.

**Table 1:** Demographic data of the individual patients and overall task performance per individual. The last column refers to the percentage of correctly identified task words (collapsed across both words). PD: parkinson’s disease (PD); TS: tourette syndrome

Case (sex)	Age	Diagnosis	Target region	Handedness	LEDD/ medication	Disease duration (years)	Speech/ language/ hearing impairments	UPDRS III (Off/ On)	Correct responses (%)
STN 1 (m)	57	PD	Bilateral STN	R	315	7	None	24/8	94
STN 2 (m)	36	PD	Bilateral STN	R	2360	8	Hypophonic speech	37/9	80
STN 3 (m)	40	PD	Bilateral STN	R / L	1710	7	None	53/24	88
STN 4 (m)	52	PD	Bilateral STN	R	400	2	Hypophonic and slurred speech	21/13	78
STN 5 (f)	51	PD	Bilateral STN	R	1197	10	None	26/18	95
STN 6 (m)	53	PD	Bilateral STN	R	1052	5	None	40/18	80
STN 7 (m)	64	PD	Bilateral STN	R	625	12	None	39/30	84
STN 8 (m)	66	PD	Bilateral STN	R	1137.5	10	None	33/ 21	64
GPi 1 (f)	19	TS	Bilateral GPi	L	Aripiprazole, Cannabidiol, delta-9-tetrahydrocannabinol	2	None		68
GPi 2 (m)	29	TS	Bilateral GPi	R	Aripiprazole	21	None		53
GPi 3 (m)	19	TS	Bilateral GPi	R	Aripiprazole	7	None		89
GPi 4 (m)	54	Dystonia	Bilateral GPi	R	Metoprolol, amitriptyline, atorvastatin, citalopram, acetylsalicylic acid, ramipril	5	None		84
GPi 5 (f)	59	Dystonia	Bilateral GPi	R	250	13	Severe dysarthria		96
GPi 6 (m)	47	Dystonia	Bilateral GPi	R	Metoprolol, ramipril	8	Mild dysarthria		87

### Experimental design, task, and stimuli

**Fig. 1:**
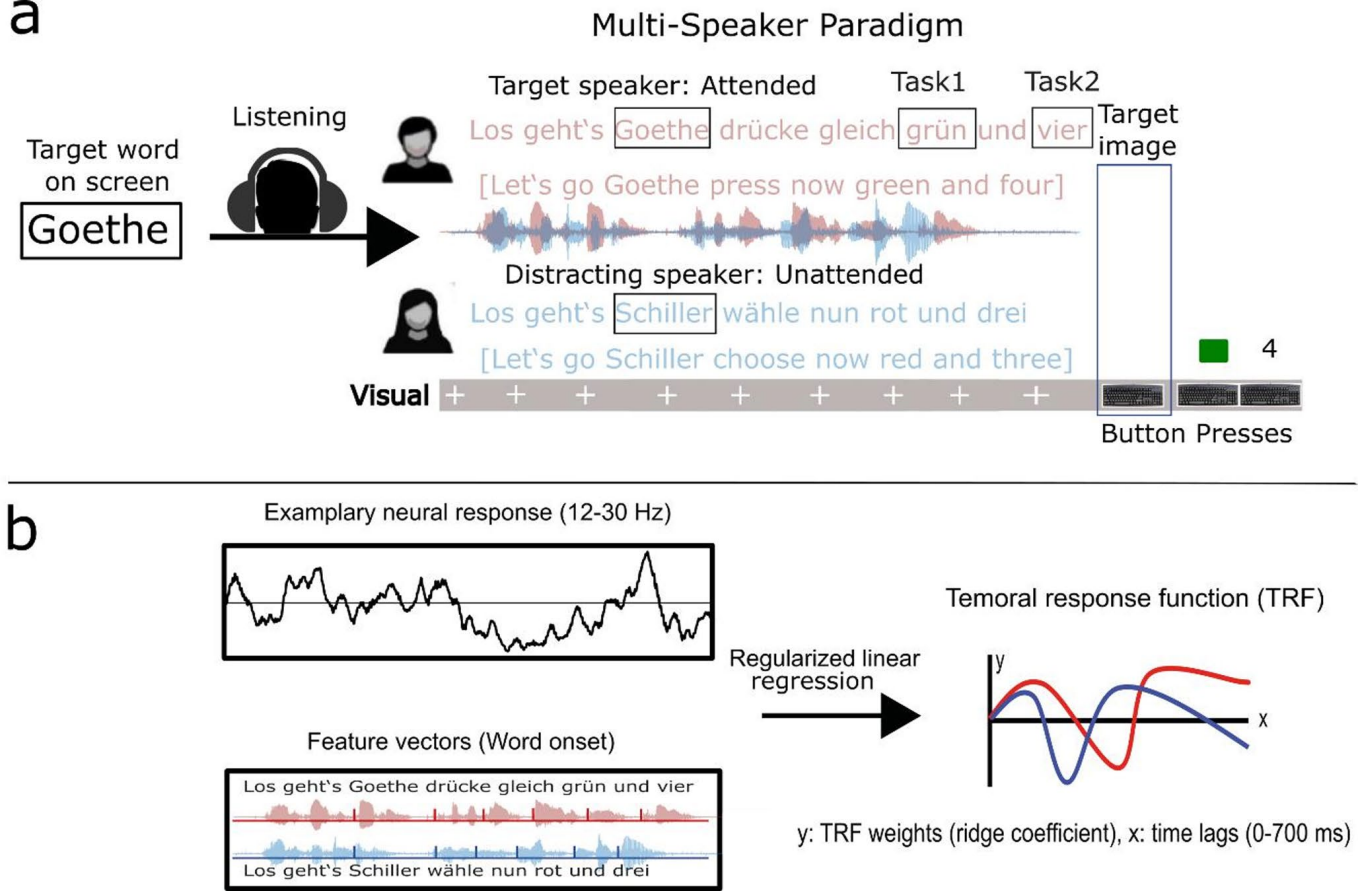
Experimental setup and selected speech features. **(a)** Trial structure of the two-speech stream paradigm. On each trial, two speech streams (male and female speakers) were presented simultaneously and binaurally to the participant. Before each experimental block, a name was printed on the video screen, indicating that the speaker who utters this name will also provide task-relevant information. An image of a keyboard was presented at the end of the sentence, indicating to participants that they should now make the responses. Upon the appearance of the target image, the participant’s task was to press the buttons referring to the color (Task1) and number (Task2) uttered in the target speech stream. **(b)** The simplified stimulus features entered into the TRF analysis consisted of two vectors. Two binary vectors were utilized as stimulus feature vectors, each corresponding to a distinct speech stream (i.e., attended and unattended). These vectors comprised zeros and ones, with “ones” denoting the word onsets. The onset of the target word, which was identified as the speakers’ names (Goethe, Lessing, Schiller, and Heine), served as the initial point for differentiating the streams based on task relevance. For the random data analysis, the word onsets in both stimulus vectors were shuffled with the restriction that the distances between the word onsets were maintained

During the performance of the experiment, patients were seated in a chair facing a video monitor (ViewSonic VG930m, 1280 × 1024 resolution, 60 Hz frame rate) at a distance of approximately 64 cm. Sounds were presented with two loudspeakers on each side of the video monitor at a comfortable volume for the patients, ensuring comprehension. We used an adapted German version of the Coordinate Response Measure Speech Corpus (Bolia et al. 2000) to represent the two-speech stream paradigm. We have applied this paradigm previously during subcortical recordings from the centromedian-parafascicular complex (Schepers et al. 2017). The trial structure of the experimental paradigm and stimulus features (word onsets) are depicted in Fig. 1a and b, respectively. All sentences started with the expression *Los geht’s* (Let’s go) followed by a target word (Goethe, Lessing, Schiller, Heine). This was followed by one of four action expressions with similar meanings, such as *drücke gleich* (press now), and a color number combination, such as *grün und drei* (green and three; Table 2). This structure resulted in sentences such as *Los geht’s Goethe entscheide dich für blau und zwei* (Let’s go Goethe choose blue and two). Importantly, in each experimental trial, two sentences were presented simultaneously and binaurally, one by a female speaker and one by a male speaker. Before each experimental block consisting of 24 sentence pairs, a target word (name) was presented in print on the video screen. The name indicated the target speaker that should be attended in the two-speaker speech stream because this speaker would later utter the task-relevant color and number word. The irrelevant, distracting speaker would always utter a different name (e.g., in the Goethe block, the distracting speaker always uttered Lessing). After the presentation of the sentences, an image of a keyboard (target image) appeared on the screen. This image indicated to the participant that she (or he) could now press the keyboard buttons corresponding to the color and the number word that the target speaker, who had uttered the target word, had pronounced. Each sentence contained one of four color words (green, blue, red, white) and one of four number words (two, three, four, five) and these words differed between the two speakers on each trial. Participants could start the subsequent trial by pressing the space bar. The keyboard image stayed on the computer screen until the participant pressed the space bar. A fixation cross appeared on the screen and remained there until the next keyboard image appeared, indicating the response interval. All sentences were root-mean-square (RMS) normalized to the same volume level. Participants were presented with 96 trials in total, except participants STN 1 and GPi 6 were presented with 72 and 85 trials due to technical reasons, respectively.

**Table 2:** Stimulus material. Multi-speaker paradigm with task-relevant auditory and visual cues

	Target word	Action expression	Task 1		Task 2	
	Goethe	drücke gleich (*press now)*	grün (*green*)		zwei (*two*)	
Los geht’s	Schiller	entscheide dich für (*your choose*)	rot (*red*)	und	drei (*three*)	
(Let’s go)	Lessing	wähle nun (*choose now*)	weiß (*white*)	(*and*)	vier (*four*)	
	Heine	nimm diesmal (*take this time*)	blau (*blue*)		fünf (*five*)	

On each trial, two simultaneously spoken sentences were presented binaurally (see Figure. 1a), where both speakers were of different genders and uttered different combinations of the sentence elements. The target word was shuffled among the four blocks. The action expression and the two task words were shuffled among all sentences. English translations for the different expressions are shown in italics. Adapted from Schepers et al. (2017)

### Electrode implantation and data acquisition

Patients were implanted bilaterally with quadripolar DBS electrodes (Medtronic 3387 or 3389 Minneapolis, MN, USA) in either the STN or GPi guided by computer tomography (CT) stereotactic surgery and microelectrode recording. Contacts on DBS electrodes were longitudinally spaced at distances of 0.5 mm in the STN and 1.5 mm in the GPi (1.27 mm diameter, 1.5 mm length). A stereotactic head frame was attached to the patient’s head under local anesthesia. Based on stereotactic CT imaging combined with preoperative MRI, the anterior (AC) and posterior (PC) commissures were identified. Post-surgical stereotactic CT scans were performed to document the placement of DBS electrodes. Electrodes were externalized for a few days prior to implantation of the pacemaker. Details of the implantation procedure and postoperative imaging have been published elsewhere (Runge et al. 2022, 2023, 2024).

Signals were sampled continuously at a rate of 2 kHz with a Digitimer D360 (Digitimer Ltd., Welwyn Garden City, Hertfordshire, UK) and digitized through a 1401 A-D converter onto a computer using the Spike2 software (both Cambridge Electronic Design, UK). During the signal recording, a 50 Hz notch filter, a high-pass filter with a cutoff frequency of 0.5 Hz, and a low-pass filter with a cutoff frequency below the Nyquist frequency were applied. Electrode impedances were below 5 kΩ during the whole recording session.

### Electrophysiological data preprocessing and analysis

The electrophysiological data of all patients were preprocessed and analyzed in MATLAB (MathWorks Inc. Natick, MA) using the open-source toolbox FieldTrip (Oostenveld et al. 2011) and customized scripts. The data concerning the minimum speech onset of the two speaker streams were examined. For automatic artifact rejection, trials were z-scored and all trials with a standard deviation ≥ 6 were removed from further analysis. This rejection criterion led to an average removal of 2.1 ± 2.4 trials (mean ± standard deviation). Of these trials, only those where participants had correctly performed the task (i.e., the two task words of the target speaker were correctly reported) were retained for further analyses. This rejection criterion led to an overall removal of 35.39 ± 30.03 trials. Electrodes were re-referenced bipolarly to their neighboring contact (three bipolar channels per hemisphere: 0–1, 1–2, 2–3) to maximize spatial selectivity and to reduce the effects of volume conduction from distant sources (Schepers et al. 2017).

For the analysis of the neural responses, the data were transformed to time-frequency space using discrete prolate spheroidal (Slepian) sequences with 2 s of window length, followed by Fourier transformation. Four tapers were applied to a frequency window from 12 to 30 Hz. Power values (squared analytic amplitude) were calculated for each data point in the trial window. A baseline was selected from − 1 to 0 s before the onset of the audio file. The average power over the baseline window was used to calculate the percent change for every data point relative to the baseline at the individual trial level. Percent change values were then averaged across trials for each time point.

### Time-frequency analysis

**Fig. 2:**
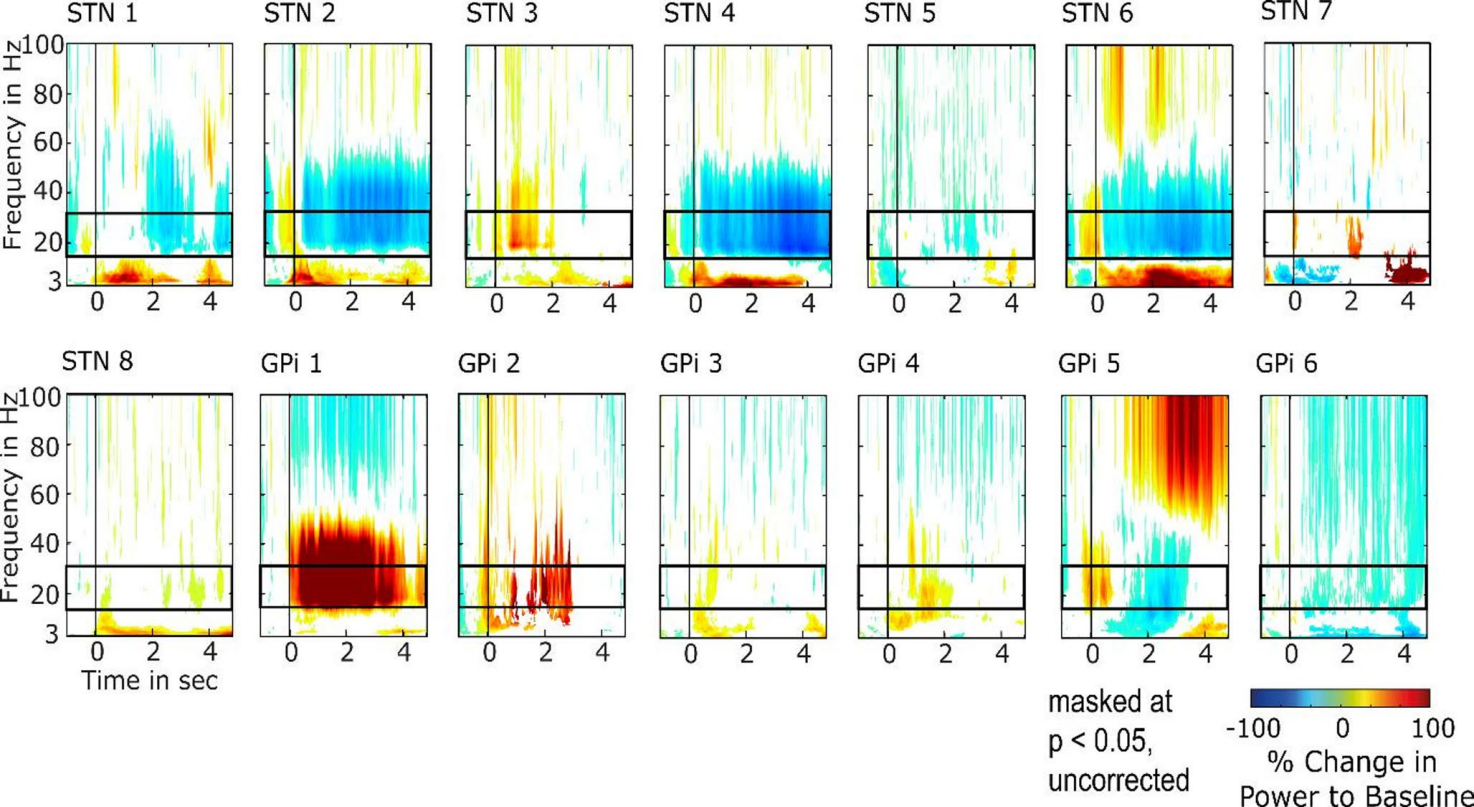
Average time-frequency response power values (squared analytic amplitude) over all trials in representative STN and GPi contacts (left hemisphere, channel 2) with sustained neural responses. The frequency band from 12–30 Hz (beta-band range) was the most consistent response across participants compared to other bands. Data are averaged across trials. 0 s refers to the onset of the target word. A baseline from − 500 to 0 ms before the speech stream onset, which occurred approximately 560 ms before the target word onset, was used

Time-frequency amplitude maps were generated for the frequency range of 3 to 100 Hz, masked at *p* < 0.05 (t-test to baseline, uncorrected), and examined across six electrode montages, three per hemisphere, for each participant (Fig. 2). The beta-band (12–30 Hz) exhibited the most consistent response across participants in comparison to other frequency bands, which is in line with a recent study demonstrating that the beta-band power of neural activity in the STN is associated with speech intelligibility (Avantaggiato et al. 2023). Moreover, Hovsepyan et al. (2023) emphasized the importance of beta-band oscillations in modulating prediction errors during speech processing, underscoring a top-down gating function that is crucial for complex auditory tasks. Furthermore, beta-band functional connectivity has been shown to support divided auditory attention during the processing of two simultaneous speech streams (Tóth et al. 2019) and is implicated in control processes (Engel and Fries 2010; Siegel et al. 2008). Thus, in light of these observations (Meyer 2018; Ortone et al. 2023), only the 12–30 Hz frequency range was selected for further investigation in the present study. For visualization purposes, uncorrected t-tests were used to threshold the maps shown in Fig. 2; however, all statistical analyses used for inference and interpretation were conducted using non-parametric tests based on a bootstrap technique.

### Word onset analysis

To determine whether the temporal structure of speech related to word onsets is represented in neural responses in the STN and GPi during sentence perception for both speech streams within a single encoding model, a regularized linear regression method was employed to estimate temporal response functions (TRFs). In this study, TRFs were used to characterize how the amplitude envelope of beta-band neural activity (12–30 Hz) encodes temporal information associated with word onsets. Beta-band oscillations were extracted from the neural signal, and their Hilbert envelope was computed to capture amplitude fluctuations over time. To ensure the robustness of the findings, TRFs were estimated separately for each participant and electrode contact using the Multivariate Temporal Response Function (mTRF) toolbox (Crosse et al. 2016), a method that predicts neural responses based on time-delayed multiple regression.

For the TFR estimation, two binary vectors, one for each speech stream (attended and unattended), served as stimulus feature vectors, consisting of zeros and ones. Here, “ones” marked the word onsets, beginning with the target word onset. The target word onset (Goethe, Lessing, Schiller, Heine) provided an initial point for differentiating the streams based on task relevance (see Fig. 1b for the experimental design). TFRs were then applied to predict neural responses as a convolution kernel with a lag length of 700 ms (0-700 ms) to the word onset time series.

**Fig. 3:**
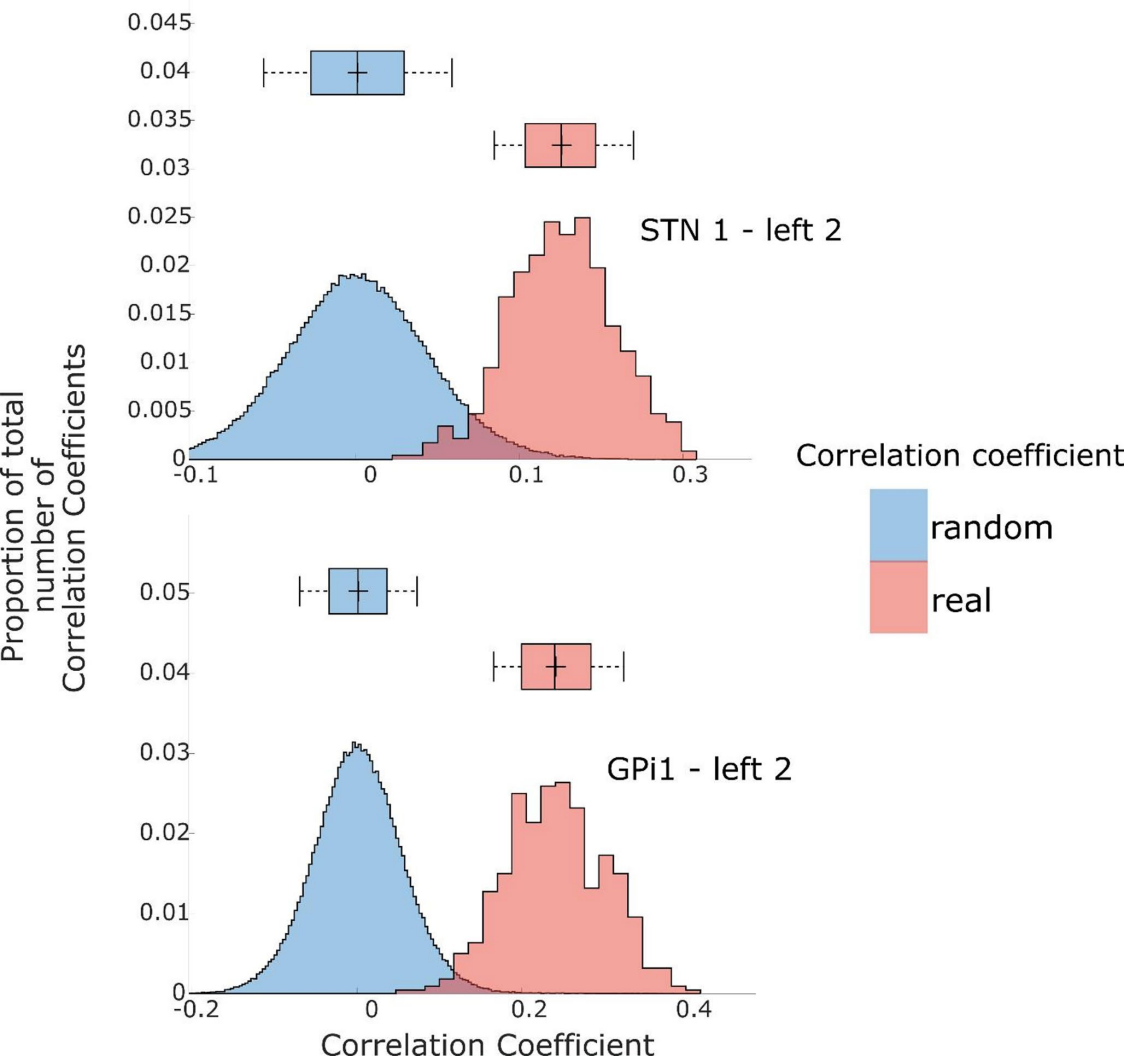
Word onsets tracking in STN and GPi. The illustration depicts exemplary significant tracking of word onsets in both STN (top) and GPi (bottom) contacts, particularly from left hemisphere channel 2. Box plots display the median, interquartile range, and range of the middle 50% of the data within the reference distribution. The x-axis represents correlation coefficients between actual and predicted neural responses, with red distributions indicating data resampled using bootstrap techniques. Blue distributions represent correlation coefficients generated from shuffled temporal structures of the speech, serving as a random reference. The y-axis represents the proportion of occurrences of correlation coefficient values within the distribution

In order to evaluate the model’s performance, cross-validation and bootstrap techniques were employed. First, the stimulus feature vector was resampled with replacement 100 times for each speech stream, generating 100 vectors that can infer information about a sample (stimulus feature vector) from resampled data. Subsequently, the bootstrap procedure was employed to ensure that the order of the word onset and its interval in resampled data was consistent with that in the sample data when estimating the distribution of generating the experimental results. Second, for each electrode contact, the resampled vectors (attended and unattended) and the corresponding neural dataset, the TRFs were estimated using five-fold cross-validation. This ensured that no test set was used in the model estimation, thus avoiding any potential bias. Then, Pearson correlation coefficients were calculated between the predicted and actual neural responses across the five folds, with the resulting values averaged to assess the model’s performance. This procedure was repeated 100 times to create a bootstrap distribution around the mean correlation, utilizing the speech’s actual temporal structure (Fig. 3, read distribution).

To assess statistical significance, a random resampling of the stimulus feature vector was conducted 500 times for each speech stream. The word onset interval positions were shuffled randomly to create a randomized temporal structure of the speech. As described above, a cross-validation schema was employed to generate a reference distribution of Pearson correlation coefficients for the randomized temporal structure, which served as a reliable baseline for comparison (Fig. 3, blue distributions). This allowed us to estimate empirical p-values for each mean Pearson correlation coefficient of the actual (real) distribution, significantly different from the random chance level.

Finally, the number of individual contact electrodes with a significant empirical p-value (*p* < 0.05) for the 48 STN contacts (2 hemispheres * 3 contacts * 8 participants) or the 36 GPi contacts (2 hemispheres * 3 contacts * 6 participants) was calculated. This calculation determined the percentage consistency of tracking the temporal structure (word onsets) of both speech streams.

### Speech stream separation analysis

In order to ascertain whether the temporal structure of attended or unattended speech related to word onsets is represented in neural responses (envelope of beta oscillation) in the STN and GPi, a linear regression model was employed again to estimate the TRFs for each speech stream separately. Subsequently, the number of individual contact electrodes with a significant empirical p-value for attended or unattended speech terms was calculated, as detailed in Sect. 2.6. The results indicated that neuronal activity in both regions can represent features of both speech streams without a consistent preference for either speaker across contacts. The full details of the results of this analysis are provided in the supplementary material.

## Results

### Behavioral performance

On average ( Table 1, last column), the two task words were correctly identified in 81% of the trials (SD: 12%, range: 53–96%), indicating that participants were attentive to the speech stimuli and demonstrated a superior ability to perform the task compared to chance, with a 6.25% probability of correct guessing per trial.

### Word-onset interval distribution

We analyzed the distribution of word-onset intervals to characterize the temporal structure of the speech stimuli. These intervals exhibited substantial variability across trials (male speaker: mean = 353 ms, SD = 134 ms; female speaker: mean = 411 ms, SD = 137 ms), corresponding to several cycles of beta-band activity. The histograms of word-onset intervals across all 96 trials are presented in Figure S1 (Supplementary Material). This variability confirms that speech onsets occurred irregularly, providing a suitable structure for assessing beta-band envelope modulations in response to naturalistic input.

### Time-frequency responses in the STN and GPi to continuous speech

Beta-band range responses were observed in both STN and GPi, with all participants displaying significant power changes compared to baseline in the 12–30 Hz frequency range during the presentation of the two-speech streams. The time-frequency responses for representative contacts in STN and GPi are illustrated in Fig. 2, masked at *p* < 0.05 (t-test to baseline, uncorrected). The power could remain constant or vary throughout the sentence presentation compared to the baseline. Some contacts showed sustained responses for the entire speech stream presentation (mean duration = 3.18 s), while others only exhibited significant power changes for a portion of the speech stimulation.

### Word onsets tracking in STN and GPi

Approximately half of the contacts in the STN and GPi demonstrated significant word onset tracking (Pearson correlation coefficients) in the 5-fold cross-validation. Specifically, 28 out of 48 STN contacts (58%) and 16 out of 36 GPi contacts (44%) exhibited significant word onset tracking in comparison to the reference distribution (*p* < 0.05). Figure 3 shows an example of the Pearson correlation coefficient distribution of actual (real; red distribution) data and random data (blue distribution) for representative individual STN and GPi contacts. These results indicate that most STN and GPi contacts tracked word onsets during speech perception.

## Discussion

Our investigation delves into the intricate role of the BG in temporal processing, specifically focusing on its contribution to encoding the temporal structure of speech. The results demonstrate that the neural responses in the STN and the GPi track word onsets during speech perception in continuous speech streams.

A substantial body of evidence from both human and animal studies has accumulated, suggesting the importance of the BG in temporal prediction and the processing of non-speech stimuli (Chiba et al. 2008; Gouvêa et al. 2015; Grahn and Rowe 2013; Kotz and Schmidt-Kassow 2015; Matell et al. 2003; Mello et al. 2015; Schwartze et al. 2015). Existing experiments utilizing non-invasive methods, such as EEG and fMRI, have touched upon the question of temporal processing in the BG (Geiser et al. 2012; Grahn 2009; Grahn and Rowe 2013; Kotz and Schmidt-Kassow 2015; Schwartze et al. 2015; Teki et al. 2011). Our study advances this understanding by employing direct electrophysiological recordings in human BG nuclei, establishing a direct link between neural responses in the beta frequency range (12–30 Hz) and the temporal structure of presented speech input. This direct relationship provides a perspective not achievable through non-invasive methods, significantly expanding our comprehension of the neural underpinnings of temporal speech processing in the human brain.

In a previous study, Gulberti et al. (2015) had conducted an EEG investigation involving patients with PD and healthy controls. The results of this study indicated a reduction in cortical beta-band oscillations in patients with PD that was correlated with predictive timing information. This finding suggests that individuals with PD experience impaired predictive timing in the cortex. Subsequent DBS targeting the STN was observed to adjust these oscillations to resemble those in healthy individuals. While this suggests a role for STN beta-band activity in supporting aspects of temporal processing, the extent to which these oscillations contribute specifically to sensory stimulus processing, like word onset, remains unclear. On the other hand, sensory inputs play a significant role in guiding attention by predicting upcoming events, and BG-centered circuits contribute to value-based decision-making (Krauzlis et al. 2014). Building upon these findings, our study demonstrates that the neural responses of BG structures reflect the temporal structure of speech.

Further insights into the BG’s role in attentional processing are provided by studies such as that by McNab and Klingberg (2008), which demonstrated a correlation between GPi activation and the storage of relevant information in working memory. GPi, as the primary output structure of the BG, might act as a gatekeeper for attended and task-relevant stimuli, enhancing stimulus processing at the cortical level. From a temporal perspective, GPi could potentially gate relevant time windows to the cortex, thus optimizing sensory stimulus processing within those temporal windows. Furthermore, GPi inactivation studies indicate its role in influencing the speed and intensity of movements without affecting goal selection (Desmurget and Turner 2008, 2010; Thura and Cisek 2017). This proposition aligns with computational models that propose the BG’s involvement in resolving conflicts between competing streams, serving as an information-routing device among cortical regions and determining the state of cortical regions (Stocco et al. 2010).

The latest research findings offer compelling evidence that, despite the motor and oscillatory dysfunctions that are associated with Parkinson’s disease, dystonia and Tourette syndrome, neuronal data from patients with these conditions can still be utilized for language and speech processing tasks (Cai et al. 2024; Yokoi et al. 2023; Schepers et al. 2017; Beck et al. 2020). Beta-band (12–30 Hz) oscillations, which are generally enhanced in Parkinson’s disease, have been implicated in a number of cognitive tasks, including speech processing and attention (Sörös et al. 2017; Jensen et al., 2013). Although these disorders affect motor control, the capacity to perceive speech remains intact due to augmented theta and beta-band activity, which enhances auditory processing and attention, respectively (Schepers et al. 2017; Beck et al. 2020). In our multi-speaker paradigm, patients exhibited the capacity to recall the target word, with an average accuracy rate of 81% in their responses. In summary, in conjunction with the present findings, these studies suggest that language and speech processing remain largely intact in these disorders and that distinctive neural signatures can be leveraged to analyze speech-related cognitive functions.

Future studies should consider simultaneous recordings from subcortical and cortical regions to elucidate further the BG’s role in speech processing and its relationship with cortical temporal processing. Building on this foundation, our study underscores the importance of investigating the precise temporal features of speech that are accurately mirrored in BG neural activity to enhance our understanding of the neural circuitry underlying speech processing.

One limitation of most LFP studies, including our own, is the selection of electrode contacts (channels) for analysis (Beck et al. 2020). In the present study, no channel selection was made for the LFP. The study utilizes bootstrap and randomization techniques to examine all montages with varying electrode contacts (0–1, 1–2, 2–3). Such an analysis would be devoid of a priori assumptions regarding the electrode contact at which task effects manifest while maintaining sensitivity to differences in electrode contact and effects across participants (Alonso-Prieto et al. 2015). The employment of bootstrapping in this context offers several advantages over parametric approaches for estimating the reliability of brain activity (Fabiani et al. 1998). First, parametric statistics typically require robust assumptions about the data distribution. However, verifying these assumptions can be difficult, especially with limited sample sizes, where violations frequently occur. Collecting data from populations with DBS is also constrained, making it challenging to obtain large samples. Additionally, there may be inherent limitations to specific experimental paradigms or procedures.

The variability in word-onset intervals demonstrates that the speech stimuli lacked strict rhythmicity, indicating that beta-band tracking in STN and GPi cannot be attributed to entrainment by periodic input. Instead, our findings suggest that beta-band envelope fluctuations reflect dynamic neural encoding of temporally structured speech. This interpretation is further supported by our randomization analysis, which showed no predictive power when the temporal structure of the speech was disrupted, highlighting the importance of the specific timing of word onsets in shaping neural responses. Moreover, the TRF analysis employed a lag window of 0–700 ms, aligning with the timescale of word-onset variability and with the expected latencies of cortical and subcortical neural responses. This lag structure enables the model to capture temporally specific neural tracking of speech without relying on assumptions of rhythmic entrainment.

Timing disruptions in speech-related STN activity have been previously reported in PD patients with speech disorders (Tankus et al. 2021). These alterations manifested as delayed and more variable neuronal responses, varying as a function of task (production, perception, or imagery) and response type (planning vs. feedback). The reported heterogeneity underscores the complex and context-sensitive nature of STN dynamics in speech processing. Although two PD patients had documented mild speech disorders (hypophonia), these were not clinically prominent features within the cohort, as evidenced by their percentage of correctly identified task words from two speaker streams (accuracy: 80% and 87%). Thus, combining the patients’ clinical profile with their percentage of correct responses enables investigation of STN dynamics in relatively intact speech function. Moreover, our approach differs methodologically, employing temporal receptive field (TRF) modeling to quantify prediction accuracy over time-lagged windows. Tankus et al. (2021) did not use TRF-based methods, their findings suggest that increased response variability, particularly in feedback-related neurons, could adversely affect model predictability. While longer TRF lags can accommodate slower neural responses, substantial temporal variability may still constrain model performance. This suggests a critical need for future studies linking trial-to-trial variability with modeling outcomes. Additionally, while Tankus et al.‘s study focused exclusively on PD patients, our cohort included individuals with PD, dystonia, and Tourette syndrome. However, it is important to note that STN recordings in our study were obtained exclusively from PD patients, none were obtained from patients diagnosed with dystonia or Tourette syndrome. Future studies that integrate STN recordings across the latter patient populations may elucidate both convergent and divergent temporal dynamics in STN activity. This could be an important direction for research that extends beyond the scope of the present studystudy.

## Conclusion

Our study lends further support to the concept that the BG are involved in temporal processing, including complex stimuli such as speech patterns. However, the specific temporal features of speech streams mirrored in BG neural activity to enhance temporal processing in cortico-basal ganglia-thalamocortical circuits remain unknown. Our findings underscore the importance of further investigating the BG’s involvement in speech processing, emphasizing the need to also consider subcortical structures to understand the neural circuitry underlying speech processing.

## Electronic supplementary material

Below is the link to the electronic supplementary material.

Supplementary Material 1

## Data Availability

No datasets were generated or analysed during the current study.

## Abbreviations

STN: Subthalamic nucleus
GPi: Globus pallidus internus
DBS: Deep brain stimulation
LFP: Local field potential
TRF: Temporal response function
