# Supplementary material for: Word onset tracking in neural responses of human basal ganglia nuclei
Source: Brain Struct Funct. 2025 Jun 25;230(6):105. doi: 10.1007/s00429-025-02968-8 (PMC12198293; doi:10.1007/s00429-025-02968-8)
Supplement: Supplementary file 1 — Supplementary Material 1 [file 429_2025_2968_MOESM1_ESM.docx]

**Word onset tracking in neural responses of human basal ganglia nuclei**

Arkan Al-Zubaidi (ORCID 0000-0002-5893-2527)^1,2,*,^, Inga M. Schepers^1^, Anne-Kathrin Beck^3,4^, Kerstin Schwabe^2,3^, Joachim Runge^3^, Mahmoud Abdallat^5^, Joachim K. Krauss^2,3^, Jochem W. Rieger^1,2,*^

^1^Applied Neurocognitive Psychology Lab, Oldenburg University, Oldenburg, Germany

^2^Cluster of Excellence Hearing4all, Oldenburg University, Oldenburg, Germany

^3^Department of Neurosurgery, Hannover Medical School, Germany

^4^Institute of Legal Medicine, Hannover Medical School, Germany

^5^ Department of Neurosurgery, University Of Jordan, Jordan

*Correspondence to:

Department of Psychology, Faculty VI, Oldenburg University, 26129 Oldenburg, Germany.

E-mail addresses: arkan.al-zubaidi@uni-oldenburg.de (Arkan Al-Zuabidi), jochem.rieger@uni-oldenburg.de (Jochem W. Rieger).

**Results**

**Speech Stream Separation in STN and GPi:**

To assess whether neural responses in the subthalamic nucleus (STN) and internal segment of the globus pallidus (GPi) selectively track attended versus unattended speech, we estimated temporal response functions (TRFs) using separate regressors for word onsets of the target and distractor speakers, separately. We then computed empirical *p*-values for each electrode contact based on a nonparametric resampling procedure, as described in section 2.6 in the manuscript.

Our analysis revealed that 48% (23 out of 48) of STN contacts and 58% (21 out of 36) of GPi contacts showed significant tracking of the target (attended) speaker. A different subset significantly tracked the distractor (unattended) speaker: 33% (16 out of 48) of STN contacts and 39% (14 out of 36) of GPi contacts. No contact demonstrated significant tracking of both streams. Neural tracking was therefore heterogeneous across contacts. Some contacts exhibited significant tracking of the target speech stream, others tracked only the distractor stream, and a subset showed no significant tracking of either. These results indicate that neural activity in both structures can encode temporal features of both competing speech streams, without showing a systematic preference for the attended stream across electrode contacts.

| 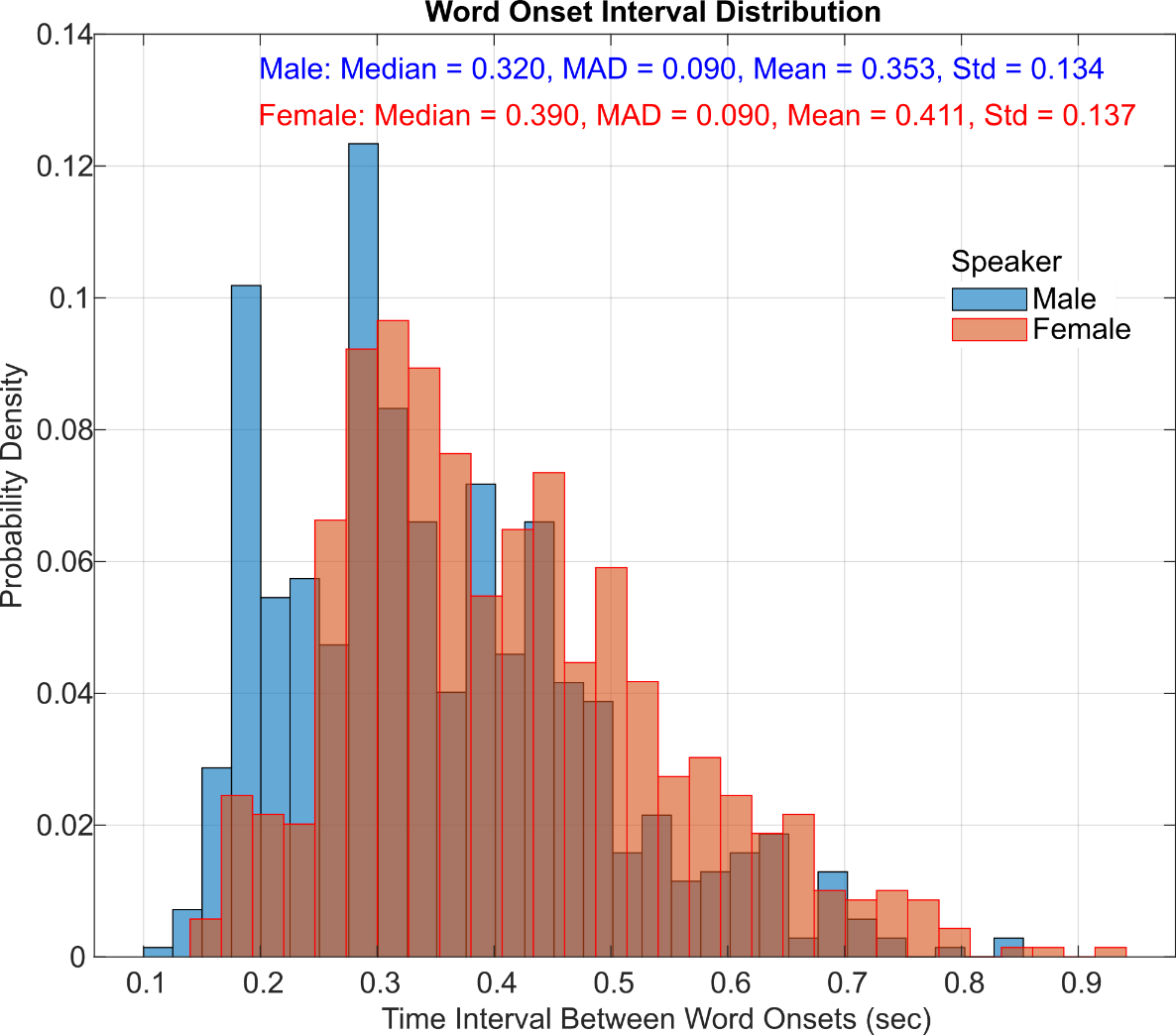 |
| --- |
| Figure S1: Word-Onset Interval Distribution across all 96 trials for male and female speakers. The x-axis represents the onset-to-word interval for each word in the male and female speech streams across all 96 trials. The y-axis shows the normalized probability, where the height of each bar reflects the relative frequency of observations (number of observations in the bin divided by the total number of observations). The sum of the bar heights is less than or equal to 1, indicating the total normalized probability across all bins. MAD refers to the median absolute deviation from the median of the data. Std refers to the standard deviation from the mean of the data. |
